# Health care providers’ perception of the frequent emergency department user issue and of targeted case management interventions: a cross-sectional national survey in Switzerland

**DOI:** 10.1186/s12873-020-00397-w

**Published:** 2021-01-07

**Authors:** Oriane J. Chastonay, Melissa Lemoine, Véronique S. Grazioli, Marina Canepa Allen, Miriam Kasztura, Joanna C. Moullin, Jean-Bernard Daeppen, Olivier Hugli, Patrick Bodenmann

**Affiliations:** 1 Faculty of Biology and Medicine, University of Lausanne, Lausanne, Switzerland; 2 Department of Vulnerabilities and Social Medicine, University Center for General Medicine and Public Health, Lausanne, Switzerland; 3 Faculty of Health Sciences, Curtin University, Perth, Australia; 4 Department of Addiction Medicine, University Hospital, Lausanne, Switzerland; 5 Emergency Department, University Hospital, Lausanne, Switzerland

**Keywords:** Frequent user, Emergency department, Case management, Health professionals’ perception, Implementation, Acceptability, Equity, Evidence-based practice

## Abstract

**Background:**

Frequent users of emergency departments (FUEDs) (≥5 ED visits/year) represent a vulnerable population with complex needs accounting for a significant number of emergency department (ED) consultations, thus contributing to EDs overcrowding. Research exploring ED staff perceptions of FUEDs is scarce.

**Objectives:**

The current study aimed to evaluate in ED staff a) the extent to which FUEDs are perceived as an issue; b) their perceived levels of knowledge and understanding of FUEDs; c) levels of perceived usefulness of case management (CM) and interest in implementing this intervention in their ED service.

**Methods:**

Head physicians of the EDs at all public hospitals in Switzerland (of various level of specialization) were sent a 19-item web-based survey, pilot tested prior to its dissemination. The head physicians were asked to forward the survey to ED staff members from different health professional backgrounds.

**Results:**

The hospital response rate was 81% (85/106). The exploitable hospital response rate was 71% (75/106 hospitals) including 208 responding health professionals. Issues and difficulties around FUEDs were perceived as important by 64% of respondents. The perceived frequency of being confronted with FUEDs was higher among nurses in more specialized EDs. In total, 64% of respondents felt poorly informed about FUEDs, nurses feeling less informed than physicians. The understanding of FUEDs was lower in the French-Italian-speaking parts (FISP) of Switzerland than in the German-speaking part. Eighty-one percent of respondents had no precise knowledge of FUED-related interventions. The perceived usefulness of CM interventions after receiving explanations about it was high (92%). However, the overall level of interest for CM implementation was 59%. The interest in CM by physicians was low across all regions and ED categories. Nurses, on the other hand, showed more interest, especially those in EDs of high specialization.

**Conclusions:**

The majority of ED staff reported being confronted with FUEDs on a regular basis. Staff perceived FUEDs as a vulnerable population, yet, they felt poorly informed about how to manage the issue. The majority of ED staff thought a CM intervention would be useful for FUEDs, however there appears to be a gap in their desire or willingness to implement such interventions.

**Supplementary Information:**

The online version contains supplementary material available at 10.1186/s12873-020-00397-w.

## Background

Frequent users of emergency departments (FUEDs) (≥5 ED visits/year) represent a small group of heterogeneous and vulnerable patients that account for a significant number of emergency department (ED) consultations, thus contributing to ED overcrowding [1–3]. In a 2016 national report from Switzerland, a country with universal access to care, FUEDs represented 2% of EDs patients and 9% of EDs consultations [4], somewhat higher percentages being reported by authors from the French-speaking-part of Switzerland (4.4% of patients and 12.1% of ED consultations) [5].

Important research efforts have been dedicated over the last 3 decades to better understand FUEDs characteristics and develop tailored interventions to address their specific needs. Research findings document that compared to occasional ED patients, FUEDs are more frequently affected by social difficulties, multiple chronic diseases, mental health disorders and/or substance abuse problems [5–7]. In response to these often cumulate vulnerabilities and need for services, research has focused on developing and testing case management (CM) interventions tailored to the needs of this population. CM interventions aim to reorient FUEDs to specific services within the hospital and community-based settings; this approach aims to improve the quality of care these patients receive and reduce ED overcrowding [8]. As reported in several systematic reviews, CM interventions have shown promising results in decreasing ED visits [8–10]. Numerous studies have also documented a positive impact of CM interventions targeting FUEDs such as lowering the number of ED visits [11], reducing costs [12] and on the health behaviors of FUEDs and their social integration [13].

Little attention has been devoted to ED staff perceptions of FUEDs, even though it may influence the care provided to FUEDs. In fact, regarding FUEDs management, a key factor in better responding to their health needs is the attitude, involvement and motivation of ED staff [14]. Attitudes of health care professionals towards patients influence the quality of care, treatment outcomes and patients’ feelings of empowerment [15, 16]. Health professionals’ perceptions of their patients depends on a variety of factors including their personal value system, previous experiences, mood, stereotypes conveyed by society, as well as work related stress (time pressure, organizational aspects, high demand for treatment) [17, 18].

Very few studies have explored ED staff perception of FUEDs. Malone [19], in her qualitative study, reported that nurses experienced a wide range of difficulties related to the perception of an existing mismatch between the demands of FUEDs and the EDs medico-clinical orientation, resulting in a feeling of failure and affecting their morale. Similarly, a recent pilot qualitative survey in French-speaking Switzerland reported that EDs staff experienced numerous difficulties related to the management of FUEDs suggesting that they may need tailored training, support and resources to address FUEDs’ specific and complex needs [20]. This has also been addressed in several case reports [21, 22]. Greater understanding of ED staff perceptions of FUEDs is needed to better delineate the kinds of support and trainings ED staff need to help them address the complex needs of this vulnerable population [23]. In response, the current study aimed to explore ED nurses and physicians’ perceptions of FUEDs, their understanding of FUEDs characteristics as well as their knowledge and their level of interest in CM interventions.

There has been a call from health authorities and hospitals in Switzerland for better care of FUEDs via interventions specifically adapted to them, such as the CM [24]. The importance of the FUEDs issue in Switzerland, as reported in various regional surveys [5, 25–27] and the scant information on the perceptions of local ED professionals [20] prompted us to conduct a survey at the national level among ED staff (medical doctors and nurses from different linguistic regions and EDs specialization levels) in order to evaluate: a) the extent to which FUEDs are perceived as an issue; b) their perceived levels of knowledge and understanding of FUEDs; c) levels of perceived usefulness of CM and interest in implementing this intervention in their ED service. Our study focused on the above mentioned aspects within a larger ongoing research project.^1^

## Methods

### The survey

A cross-sectional national survey addressed to the ED head-physicians of all public hospitals in Switzerland was sent between September 2017 and March 2018. The survey was limited to general emergency services open 24 h a day, 365 days a year accepting adult patients (*n* = 106 hospital sites). The survey was hosted on a web-based platform (Survey-Monkey) and consisted of 19 closed-questions in three (German, French, Italian) of the four national languages. The survey consisted of questions relating to ED staff perception of FUEDs as well as their perception and interest in implementing CM interventions tailored to FUEDs. The final questionnaire was sent along with a short explicative text about FUEDs (defined as ≥ 5 ED visits/year) and CM interventions (defined as interventions that consist of coordinating care in collaboration with the patients’ healthcare network, while providing individualized support that aims to strengthen their resources and skills). The ED head physicians were asked to forward the survey to ED colleagues from different professions (head nurses, nurses, chief residents). At week two, three and four, an e-reminder was sent to the receiver of the first e-mail. Two telephone calls were subsequently made to the non-respondents either to the ED head-physician in person or to his/her secretary.

### The questionnaire

The questionnaire was developed by a panel of professionals involved in research and care of FUEDs as well as in CM implementation and evaluation, i.e. ED clinicians, public health professionals, community health nurses, clinical researchers. The panel conducted brainstorming sessions to develop a set of items intended to estimate ED staffs’ perception of FUEDs and their knowledge of FUEDs characteristics (i.e. the extent to which they were familiar with characteristics commonly attributed to FUEDs [6, 26, 28–30]) as well as their perception of the usefulness of CM interventions and their interest in implementing CM programs.

The questionnaire was reviewed and approved by an ED staff panel (3 clinicians, an epidemiologist and a psychologist). The reviewed questionnaire was pilot tested by 14 health professionals, including 7 medical doctors, 6 nurses and one psychologist, all of them not involved in the survey prior to its dissemination. The questionnaire is provided in Additional file 1.

### The explanatory variables

In Switzerland, EDs are categorized in 3 groups: Category 1 presents the highest level of specialization (> 20′000 emergency consultations per year, presence of a senior medical doctor 24 h/day, Swiss Society of Emergency and Rescue Medicine (SSERM) certified ED, meeting three out of four of the following criteria: located in a trauma centre, having access to an intensive care unit, a stroke unit, a cardiac catheterization laboratory, and at least 50% of the nursing staff specialized in emergency medicine), category 2 (> 9′000 emergency consultations per year, SSERM certified ED, presence of a senior medical doctor 24 h/day, an intensive care unit and at least 25% of nurses specialized in emergency medicine), and category 3 presenting a more basic level.

In our study, 72% of the contacted EDs (*n* = 106) were located in the German-speaking part (GSP) (*n* = 76), 21% in the French-speaking part (*n* = 22) and 7% in the Italian-speaking part (*n* = 8) of Switzerland.^2^ Given the low percentage of Italian-speaking participants and their common Latin roots, their results were pooled with those from the French-speaking participants under the category French-Italian-speaking part (FISP) for statistical analyses. In terms of the GSP 11% of EDs were category 1, 17% category 2 and 72% category 3; in the FISP 17% of EDs were category 1, 20% category 2 and 63% category 3.

### The statistical analyses

All statistical analyses were performed with R 3.5.3 (www.r-projet.org).

The ED attrition according to ED category and linguistic region was investigated using the probability to collect at least one exploitable response from health professionals with a logistic regression including the interaction between ED category and linguistic region. The number of responses by ED, according to ED category and linguistic region, was analyzed using a truncated Poisson distribution with the package VGAM (www.stat.auckland.ac.nz/~yee/VGAM) and type III ANOVA tests. The details of the attrition data are provided in Additional file 2.

Response variables collected from health professionals were analyzed with the packages lme4, afex and glmmTMB using generalized linear mixed models including the hospital site as a random effect to take into account the homogeneity of responses among professionals working in the same ED. Linguistic region, ED category and profession were included as fixed effects to estimate the mean effect of each of these explanatory variables as well as their interactive effects, and to adjust for bias linked to unbalanced design at the three levels, i.e. individual, site and regional level (see comments under Tables 3 and 4) [31].

The response variables were coded to follow a binomial or a centered normal distribution, except the number of known interventions that followed a zero-inflated Poisson distribution. This coding allowed to interpret the estimates of the final statistical models as a lower /greater probability than by chance for binary variables and a negative/positive perception for normally distributed variables where zero reflects an absence of individual or collective agreement. Because the aim was to measure the average perception over all groups as well as within groups, the explanatory variables were numerically coded (e.g., 1 for doctors and − 1 for nurses) and analyses were weighted for sample size within groups to provide unbiased estimates of population parameters.

Missing data were investigated over 25 items and were replaced by zero for the agreement scales (i.e., absence of agreement). Variation in sample size among response variables reflects the missing data structure. The analyzed variables are outlined in Additional file 3.

## Results

### Descriptive statistics

Overall, 208 health professionals (113 physicians and 95 nursing staff) answered the questionnaire: 110 from GSP and 98 from FISP; 42, 62 and 104 from EDs of category 1, 2 and 3 respectively.

The majority (58.4%) of health professionals reported daily or weekly admissions of FUEDs. According to 20.6% of ED staff, FUEDs accounted for less than 5% of all ED consultations, for 44.5% of respondents FUEDs represented 5 to 10% of all ED consultations, and for 17.8% of respondents 11 to 20%, and for 17.1% of respondents 30% or more. Yet, 79.4% of respondents mentioned that they were not aware of a specific tool/indicator in their respective hospital allowing the precise measurement of the frequency of FUEDs consultations. FUEDs were perceived as an important issue by 63.9% of respondents. Sixty-four percent felt not well informed about FUEDs*.* When asked about the characteristics of FUEDs, over 75% recognized that FUEDs represented a high vulnerability group, often suffering from chronic disease or psychiatric disorders, feeling discriminated and of disadvantaged background. Statistically significant differences existed among nurses and medical doctors for several of these characteristics (see Table 1). For example, physicians endorsed the following characteristics of FUEDs more frequently than nurses: *often of disadvantaged backgrounds*, 89.1% physicians versus 71.6% nurses (*p*< 0.005); *often feeling discriminated*, 86.0% physicians versus 68.4% nurses (p< 0.005); *often presenting psychiatric disorders* 89.5% versus 63.9% (*p*< 0.001). Globally, 81.0% of respondents had no knowledge of FUED-related interventions. When asked about the potential usefulness of CM interventions in their respective hospitals (a definition of which had been given in the questionnaire), 46.6% of respondents considered that a CM intervention would be very or extremely useful and 46.1% said it would be rather useful. The potential impact of CM interventions, as perceived by ED staff, is shown in Table 2. As an example, nurses considered several items as more useful than physicians: *CM insure better targeted response to needs of FUEDs* 84.0% versus 69.9% (*p*< 0.01); *CM facilitate the collaboration with community/primary care* 85.0% versus 64.7% (p< 0.001). According to 24.0%, a specific person in their hospital was designated as the case manager dedicated to FUEDs; 58.5% of ED staff were willing to be personally involved in the development and implementation of a CM team in their respective hospital and 42.8% to be directly involved in such a team*.*

**Table 1:** FUEDs characteristics as perceived by ED health personnel according to profession on a 0 to 10 agreement-scale (zero: totally disagree; six: agree; 10: totally agree)

FUEDs characteristics	*N* total respondents	% of total personnel agreeing	% of medical doctors agreeing	% of nursing personnel agreeing	*P* value
Inappropriate use of EDs	*n*: 193	90.7%	90.2%	91.3%	ns
High social and medical vulnerability	*n*: 192	87.1%	91.0%	82.8%	ns
Suffering from chronic disease	*n*: 196	82.1%	82.2%	82.1%	ns
Often of disadvantaged backgrounds	*n*: 194	80.4%	89.1%	71.6%	< 0.005
Feeling often discriminated	*n*: 195	77.4%	86.0%	68.4%	< 0.005
Presenting psychiatric disorders	*n*: 128	77.4%	89.5%	63.9%	< 0.001
Patient has no general practitioner	*n*: 198	74.2%	68.9%	80.1%	ns
Living near an emergency service	*n*: 197	59.4%	54.4%	54.2%	ns
Mostly foreigners	*n*: 198	51.9%	60.8%	42.8%	0.01
High mortality rates	*n*: 173	37.6%	35.8%	39.4%	ns

**Table 2:** Perceived potential usefulness of specific CM interventions targeting FUEDs by ED health personnel according to profession on a 0 to 10 agreement-scale (zero: totally disagree; six: agree; 10: totally agree)

Potential usefulness	*N* total respondents	% of total personnel agreeing	% of medical doctors agreeing	% of nursing personnel agreeing	*P* value
Support teams facing complex medico-social situations	*n*:199	77.4%	76.7%	78.1%	ns
Insure better targeted response to needs of FUEDs	*n*:199	76.9%	69.9%	84.0%	0.01
Facilitate the collaboration with community/primary care	*n*:196	74.5%	64.7%	85.0%	0.001
Decrease the number of FUEDs emergency room visits	*n*:198	69.6%	66.0%	73.7%	ns
Spend less time on low-level emergency patients	*n*:199	58.3%	60.2%	56.2%	ns
Spend less time on patients with complicated situations	*n*:197	37.0%	35.0%	39.4%	ns

### Analytical statistics

The overall summary of the different components ED staff perceptions to FUEDs and CM interventions are summarized in Table 3. The influence of the three levels investigated, i.e. health care profession, ED category and linguistic region is summarized in Table 4.

**Table 3:** Overall mean of perception and interest in FUEDs issues and related interventions

Response variables	Overall mean	Est.	SE	*z/t*-value	Est. Df	*P*-value
Question: How often have you been confronted with FUEDs over the past 2 years? *Probability of being confronted with FUEDs every week or day (*Awareness of FUEDs)	**0.59**	0.37	0.18	2.13	–	**0.033**
Question: How important is the FUEDs problem in your hospital? *Probability of FUEDs being an issue in the ED* (Perception of FUEDs)	**0.67**	0.73	0.21	3.52	–	**< 0.001**
Question: How do you evaluate your level of knowledge on the FUEDs issue? *Perceived knowledge of FUEDs from not at all (−2) to very familiar (2)*	**−0.32**	−0.32	0.09	−3.57	43.96	**< 0.001**
Question: How do you evaluate your level of knowledge on CM interventions? *Perceived knowledge of CM from not at all (− 2) to very familiar (2)*	**− 0.76**	−0.76	0.09	−8.67	37.96	**< 0.001**
Question: In your opinion, what are the characteristics of FUEDs? *From totally disagree (−5) to totally agree (5)* (Understanding of FUEDs)	**1.36**	1.36	0.08	16.74	56.59	**< 0.001**
Question: Which of these interventions do you know? *How many interventions are known (from 0 to 5)* (Knowledge of FUEDs interventions)	**1.77**	0.57	0.16	3.45	–	**< 0.001**
Question: Which of these interventions do you know? *Probability of knowing at least one intervention* (Knowledge of FUEDs interventions)	**0.21**	1.29	0.22	5.89	–	**< 0.001**
Question: To what extent do you think an intervention would be useful in your hospital? Perceived usefulness of interventions *from not useful at all (−5) to extremely useful (5)*	**1.14**	1.14	0.13	8.83	194.00	**< 0.001**
Question: To what extent do you think a CM intervention targeting FUEDs would be useful in your department? Perceived usefulness of CM *from totally disagree (−2) to totally agree (2)*	**1.29**	1.29	0.08	16.89	42.21	**< 0.001**
Question: To what extent would you be interested in supporting/participating in implementing a case management intervention? Interest in CM implementation *from a full disinterest (−5) and a full interest (5)*	0.10	0.10	0.19	0.50	190.00	0.615

Overall mean: transformed estimate of the statistical model intercept when necessary; *Est* estimate of the model intercept, *SE* standard error of the model intercept, *z/t*-value *z*-value for Count distribution (Binomial or Poisson distribution) or *t*-value for Normal distribution, *Est. Df* estimated number of freedom degree for general linear mixed models (Normal distribution); *P*-value: statistically significant different from by chance (probability of 0.50) for Binomial distribution or zero for Normal distribution. In bold are highlighted the estimates that are statistically significant

The estimation of intercept was done under the restricted maximum likelihood with GLMMs including the three mean effects: health care profession, *ED* category and linguistic region, all standardized (i.e., of mean zero and variance 1) in order that the (transformed) intercept represent the mean of a balanced sample of physicians and nurses within ED category and within linguistic region referred as the overall mean. The knowledge of FUEDs interventions was analyzed with a zero-inflation Poisson distribution leading to the estimation of two coefficients: the average number of events per interval (for the Poisson distribution) and the probability of extra zeros (*p*) that can be used to approximate the probability of knowing at least one intervention (1-p)

**Table 4:** Influence of health care professions, ED categories and linguistic regions on awareness, perception, knowledge of and interest in FUEDs issues and related interventions

Response variables	Interactions			Profession		ED category		Ling. Region	
	Name	LRT*χ*^2^	*P*	LRT*χ*^2^	*P*	LRT*χ*^2^	*P*	LRT*χ*^2^	*P*
Question: How often have you been confronted with FUEDs over the past two years? (Awareness of FUEDs)	ED category x profession	**5.14**	**0.023**	–	–	–	–	0.74	0.389
Question: How important is the FUEDs problem in your hospital? (Perception of FUEDs)	all	> 2.13	> 0.143	0.95	0.330	**16.41**	**< 0.001**	0.47	0.494
Question: How do you evaluate your level of knowledge on the FUEDs issue? (Perceived knowledge of FUEDs)	all	> 2.73	> 0.095	**4.14**	**0.042**	**5.11**	**0.024**	2.06	0.152
Question: How do you evaluate your level of knowledge on CM interventions? (Perceived knowledge of CM)	all	> 0.85	> 0.355	2.10	0.147	2.49	0.115	3.59	0.058
Question: In your opinion, what are the characteristics of FUEDs? (Understanding of FUEDs)	all	> 2.24	> 0.135	2.72	0.099	0.04	0.832	**30.49**	**< 0.001**
Question: Which of these interventions do you know? (Knowledge of FUEDs interventions - Zero-inflation model)	all	> 1.19	> 0.275	**4.59**	**0.032**	0.53	0.467	0.14	0.707
Question: Which of these interventions do you know? (Knowledge of FUEDs interventions - Count model)	all	> 2.01	> 0.156	1.73	0.189	1.89	0.169	1.15	0.283
Question: To what extent do you think an intervention would be useful in your hospital? (Perceived usefulness of interventions)	all	> 2.72	> 0.095	1.53	0.216	**19.50**	**< 0.001**	**18.51**	**< 0.001**
Question: To what extent do you think a CM intervention targeting FUEDs would be useful in your department? (Perceived usefulness of CM)	all	> 3.27	> 0.070	1.79	0.181	**7.77**	**0.005**	0.32	0.572
Question: To what extent would you be interested in supporting/participating in implementing a case management intervention? (Interest in CM implementation)	ED category X profession	**16.44**	**< 0.001**	–	–	–	–	**6.58**	**0.01**

Bold highlights the statistically significant effects. All models are GLMMs with hospital site as a random effect. Model selection was done under maximum likelihood using as initial model the model including the three-way interaction (and all two-way interactions) among the mean effects: health care profession, ED category and linguistic region. Health care profession and linguistic region, with two groups, were coded as categorical variables while ED category, with three groups, was coded as a linear variable to increase statistical power and simplify model interpretation. Interactions were eliminated using a backward stepwise approach using likelihood ratio tests (LRT*χ*^2^). The final models, when interactions were non-significant, included all mean effects. Awareness of FUEDs and perception of FUEDs were analyzed with a logistic mixed regression. The knowledge of FUEDs interventions (from 0 to 5) was analyzed using a zero-inflation Poisson distribution (that breaks down the data in two models: one that models the presence of extra-zeros; i.e., zero-inflation model and one that models a Poisson distribution; i.e., the count model). All others variables were analyzed using a Normal distribution

The estimated frequency with which ED staff faced FUEDs on a weekly or daily basis, was 0.59 overall and was higher than by chance (Table 3). It varied between nurses and physicians depending on the ED category (Table 4). The estimated frequency ED staff observed FUEDs every week or day in ED category 2 was higher than by chance (probability = 0.66, z-value = 2.16, *P* = 0.031) for nurses but not for physicians (probability = 0.61, z-value = 1.92, *P* = 0.054). Moreover the estimated frequency with which they observed FUEDs on a weekly or daily basis increased from the lower level to the higher level of ED specialization (β = 1.15 ± 0.35, z-value = 3.23, *P* = 0.001) for nurses but not for physicians (β = 0.20 ± 0.27, z-value = 0.711, *P* = 0.477).

The estimated probability that FUEDs were perceived as an issue (*question: How important is the FUEDs problem in your hospital?*), approximated 0.67 overall (Table 3), but increased from the lower level to the higher level of ED specialization (β = 0.85 ± 0.22, z-value = 3.81, *P* < 0.001).

The perceived knowledge of FUEDs *(question: How do you evaluate your level of knowledge of the FUEDs issue?)* was negative overall (− 0.32 ± 0.09, *P* < 0.001, Table 3) and varied according to profession and ED category (Table 4). The perceived knowledge of FUEDs was negative for nurses (− 0.49 ± 0.13, *t*-value = − 3.89, *P*< 0.001) but approximated zero for physicians (− 0.18 ± 0.11, *t*-value = − 1.57, *P*=0.119). However the perceived knowledge of FUEDs increased from the lower level to the higher level of ED specialization (β = 0.20 ± 0.09, z-value = 2.28, *P* = 0.027). The capacity of identifying main characteristics of FUEDs (*question: In your opinion what are the characteristics [inappropriate use of EDs; high social and medical vulnerability; suffering from chronic disease; high mortality rates; patient has no general practitioner; feeling often discriminated; often of disadvantaged backgrounds; presenting psychiatric disorders; living near an emergency service; mostly foreigners] of FUEDs?*), was positive (1.36 ± 0.08, *P*< 0.001, Table 3), but varied according to the linguistic region: lower in the FISP than in the GSP (0.81 ± 0.12, *t*-value = 6.72, *P*< 0.001 for FISP and 1.85 ± 0.11, *t*-value = 16.56, *P*< 0.001 for GSP). The probability of having knowledge of interventions tailored to FUEDs was low (approx. 0.21) and the mean number of known interventions (from 0 to 5) was 1.77 (*question: Which of these interventions [case management, diversion health strategies, individual health care plan, therapeutic education, others] do you know?*). Although the number of known interventions did not vary significantly according to the profession, ED category nor regions, the probability of knowing interventions tailored to manage FUEDs approximated 0.30 for physicians and 0.14 for nurses.

The perceived knowledge of CM *(question: How do you evaluate your level of knowledge on case management intervention?)* was significantly negative (− 0.76 ± 0.09, *P* < 0.001, Table 3). It did not vary according to the profession, ED category, nor linguistic region (Table 4). The perceived usefulness of interventions tailored to manage FUEDs *(question: To what extent do you think an intervention would be useful in your hospital in order to: decrease the number of FUEDs emergency room visits; insure better targeted response to the needs of FUEDs; support teams facing complex medico-social situations; spend less time on patients with complicated situation; spend less time on low-level emergency patients; facilitate the collaboration with community/primary care?)* varied according to ED category and linguistic region (Table 4). The perceived usefulness of interventions (on a scale of − 5 to 5) approximated to 1.75 ± 0.19 (*t*-value = 9.32, *P*< 0.001) in the FISP and 0.59 ± 0.18 (*t*-value = 3.28, *P* = 0.001) in the GSP, while it increased with ED category (β = 0.62 ± 0.13, *t*-value = 4.82, *P*< 0.001). In comparison, the perceived usefulness of CM *(question: To what extent do you think a CM intervention targeting the FUEDs issue would be useful for your department?)* approximated 1.29 ± 0.08 on a scale of − 2 to 2 and increased with ED category (β = 0.21 ± 0.07, *t*-value = 2.87, *P* = 0.006).

Despite a positive perception of the usefulness of interventions and of CM in particular, the overall level of interest in CM implementation in ED *(question: To what extent would you be interested in supporting/participating in implementing a case management intervention?*) approximated zero (Table 3), but varied according to the region and the profession within ED (Table 4). On average, the level of interest for CM implementation approximated zero in the GSP (− 0.34 ± 0.25, *t*-value = − 1.33, *P* = 0.186) and was positive in the FISP (0.55 ± 0.27, *t*-value = 2.02, *P* = 0.045). Moreover, the level of interest for CM implementation approximated zero (− 0.39 ± 0.25, *t*-value = − 1.53, *P* = 0.129) and did not vary according to ED category for the physicians (β = − 0.04 ± 0.25, *t*-value = − 0.16, *P* = 0.870). In contrast, the level of interest for CM implementation was positive (0.64 ± 0.27, *t*-value = 2.33, *P* = 0.021) and increased from the lower level to the higher level of ED specialization for the nurses (β = 1.47 ± 0.27, *t*-value = 5.36, *P* < 0.001).

## Discussion

The majority of ED staff (64.3%) did not feel well informed about FUEDs, nor were they familiar (80.9%) with specific interventions aimed at reducing the number of FUEDs and improving the quality of care of these patients. This despite the finding that 63.9% of ED staff considered FUEDs to be an important issue and FUEDs were observed by 58.4% of staff on a weekly or more basis. Yet, only 42.8% of respondents were willing to introduce and integrate a team in charge of developing specific interventions (e.g. CM) targeting FUEDs.

Among the respondents of our study, 44.5% estimated that FUEDs represented 5 to 10% of their ED consultations, which is within the range reported in Switzerland (9% with regional variations), from the Federal Statistical Office’s ED outpatient data collection [4]. No significant regional variations were observed.

In our study, 63.9% of respondents considered FUEDs as an important issue. This is not a surprising finding since FUEDs often present with a *“substantial burden of disease”* (cardiovascular diseases, cancers, mental illness, multi-morbidities, behavioral health problems), as well as psycho-social problems, and increased mortality rates [32–34], making them complex to treat [35]. FUED might also contribute to ED overcrowding [2, 36], which in turn may contribute to stress and dissatisfaction of ED staff [37, 38].

Findings indicated that more than 64% of respondents did not feel well informed, nurses more so than physicians. Of interesting note, whereas most respondents claimed little knowledge of the FUEDs, they nevertheless accurately described their main characteristics as described in several reviews [1, 3, 6, 39]. Examples of FUED characteristics include social and medical vulnerability, chronic disease or psychiatric disorders [6, 29]. Taken together, our findings that ED staff seem aware of the vulnerability and complexity of these patients yet feel fairly uninformed about them raise the question of the necessity to provide them with FUED-related training. Indeed, FUED characteristics potentially exceed the resources of EDs and their staff [3, 39]. In fact, past research showed that these patients solicit “*deeper clinician involvement because of their familiarity and their often intractable medical and social problems*” [19]. Tailored FUED-training may be necessary to enhance ED staff efficiency in providing care to FUEDs via a greater knowledge base about the underlying causes of frequent emergency service use and help them face difficulties related to FUED healthcare.

Eighty-one percent of respondents had no precise knowledge of specific interventions targeting FUEDs. The probability of knowing about interventions to manage FUEDs was slightly higher among physicians than nurses. The perceived usefulness of such interventions was higher in respondents from FISP as well as staff from EDs of higher specialization. Those regional and structural differences may reflect a difference in both exposure and sensitivity to the FUEDs issue, or they may reflect different levels of organization or resources of the EDs, as some authors have suggested [40].

The perceived usefulness of CM interventions was high across the board (1.29 +/− 0.08 on a − 2 to + 2 scale), increasing with ED specialization. This appears to be a good sign for implementing a CM intervention in EDs across Switzerland. The effectiveness of CM interventions targeting FUEDs has been assessed in several thematic and systematic reviews [9, 18, 41, 42]: a decrease of ED visits, and a possible reduction of costs are reported as well as a better response to the health needs of these patients and a reduction of social problems. The key to the success of such interventions is a close partnership with community health services, a tight coordination of care, a continuous patient support throughout their care path, appropriate training of ED staff and case managers, and staff maintaining focus on FUEDs’ needs [43, 44]. Up to 61% of FUEDs “*stated the primary reason for their visit was that they felt that their health problem could only be treated in an ED”* and 53% found “*that having a nurse to work with you one-on-one to help manage health care needs would be most helpful in achieving optimal health*” [44].

The overall level of interest for CM implementation approximated zero on our scale (0.1), which reflects a neutral position between totally disinterested and totally interested, meaning that a small majority appeared interested in such an approach. The lack of enthusiasm for CM implementation may be explained by the fact that health professionals in EDs often work in strained and stressful conditions that do not allow them to take on additional activities without additional resources. Indeed, the implementation of *CM* programs in health structures requires appropriate training of health professionals and reorientation of activities [45], which in turn implies additional/ complementary funding. Hudon et al. [46] expressed it as follows: *“While material, organizational and human resources bring the necessary support for providers of healthcare services in their activities and make the implementation of CM program possible, each of these resources depends on adequate public funding. Resource allocation within the CM program also have an impact on the effectiveness of the five main intervention activities: case finding; assessment; care planning; care coordination and self-management support”.*

Interestingly, results revealed differences across linguistic regions, with more interest shown in FISP than in GSP. The interest of physicians remained low across the regions and the ED categories. Nurses, on the other hand, seemed to show more interest, especially those in EDs of category 1. The latter could therefore serve as a starting point in the establishment of CM programs allowing the guidance of “*patients through the care process and provide social support*” [43].

### Limitations

Several limitations must be mentioned. First, the questionnaire was not previously validated in the literature; it was developed within a larger ongoing research project by a panel of clinicians and researchers familiar with the FUEDs and CM issues. Second, the ED head physicians were the first recipients of the survey questionnaire with the request to forward the questionnaire to ED collaborators. This process may not have been systematically done and may have led to some distortion of respondents; however this opportunistic approach was the most logistically efficient. Third, the questionnaire did not measure years of experience in respondents; we cannot therefore exclude that experience length was shorter than one year for some respondents. That said, most respondents were head nurses and physicians, which requires multiple years of previous experience. Fourth, participation was higher in the FISP region as well as in category 1 EDs, which reduces the representativeness of the overall numbers. Fifth, missing data was higher in the GSP region than in the FISP region, which may slightly distort the Swiss reality. Despite adjusting for the unbalanced sample, difference in participation rates and missing data across regions and ED category may have introduced some bias and decreased the statistical power of the study. Finally, the responses were self-reported by ED staff based on their overall experience with the FUEDs, which can result in some subjectivity.

## Conclusion

To our knowledge this is the first national survey in Switzerland exploring ED staff perceptions on FUEDs and CM interventions tailored to this population. Most ED staff members who participated in our survey report receiving FUEDs on a regular basis and perceive them as patients in a situation of vulnerability. Furthermore, the majority of ED staff would find a CM intervention targeting FUEDs useful and to a lesser extent would be willing to participate in its local implementation. CM could provide resources to meet the needs of FUEDs, by redirecting the most time-consuming aspects (psychosocial, coordination of care) of FUEDs care, and while allowing ED staff to deal with “true” emergencies. Our results reinforce the need to provide more targeted interventions for FUEDs and results are promising with regard to a wider implementation of a CM approach in Switzerland.

## Supplementary Information

**Additional file 1: Appendix 1.** The questionnaire.

**Additional file 2: Appendix 2.** Attrition and missing data.

**Additional file 3: Appendix 3.** Response variables.

## Data Availability

The datasets used and/or analyzed during the current study are available from the last author on reasonable request.

## Abbreviations

CM: Case management
ED: Emergency department
FISP: French-Italian-speaking parts
FUEDs: Frequent users of emergency departments
GSP: German-speaking part
SSERM: Swiss Society of Emergency and Rescue Medicine

## Footnotes

1 Swiss National Science Foundation SNF–PNR 74. Proposal n^o^ 407440–167341. *Implementing a case management intervention for frequent users of the emergency department: a multi-center study in Switzerland.*

2 Proportion of residents in the GSP 71%, in the French-speaking 24.5%, in the Italian-speaking part 4.5%
