# Supplementary material for: Health care providers’ perception of the frequent emergency department user issue and of targeted case management interventions: a cross-sectional national survey in Switzerland
Source: BMC Emerg Med. 2021 Jan 7;21:4. doi: 10.1186/s12873-020-00397-w (PMC7792123; doi:10.1186/s12873-020-00397-w)
Supplement: Supplementary file 1 — Additional file 1: Appendix 1. The questionnaire. [file 12873_2020_397_MOESM1_ESM.docx]

**Appendix 1: The questionnaire**

| *This short survey is part a project funded by the Swiss National Science Foundation in order to know the opinion of Emergency Department staff regarding the issue of Emergency Department Frequent Users, defined as as patients who have consulted the emergency department 5 or more times during the previous 12 months, and interventions specifically intented to tackle the issue .* |
| --- |
| **1. Which is your current position?** |
| - Nurse - Head nurse - Social worker - Head social worker - Head physician - Chief resident - Other |
| **2. In which canton do you work?** |
| - Multiple choice the list of Swiss cantons |
| **3. In which hospital do you work** |
| - Multiple choice the list of the hospitals contacted |
| **4. Is your hospital's emergency department open 24/7?** |
| - Yes - No |
| **5. What category of patients do you receive in the emergency department in which you work?** |
| - General adult emergencies - Paediatric emergencies - Gynaecological emergencies - Psychiatric emergencies - Other |
| **6. How often have you been confronted with FUEDs over the past two years?^[[1]](#footnote-1)^** |
| - Every day - Every week - Once or twice a month - Once or twice a year - Never |
| **7. Is there an indicator allowing you to quantify the FUEDs in your hospital?** |
| - Yes - No |
| **8. What proportion of your annual consultations is represented by FUEDs?** |
| - Scale in percentage (0-100%) |
| **9. How important is the FUEDs problem in your hospital?** |
| - Extremely important - Important - Somewhat important - Not important |
| **10. How do you evaluate your level of knowledge on the FUEDs issue?** |
| - Very familiar - Somewhat familiar - Not very familiar - Not at all familiar |
| **11. Are you aware of any specific interventions targeting FUEDs?** |
| - Yes - No |
| **12. Which of these interventions do you know?** |
| - Case management - Diversion health care strategies (not urgent patients being redirected to other primary health care services) - Individual health care plan - Therapeutic education - Other |
| **13. In your opinion, what are the characteristics of FUEDs?** |
| - Inappropriate use of EDs - High social and medical vulnerability - Suffering from chronic disease - High mortality rates - Patient has no general practitioner - Feeling often discriminated - Often of disadvantaged backgrounds - Presenting psychiatric disorders - Living near an emergency service (<10km) - Mostly foreigners - Likert scale (0: totally disagree; 10: totally agree) |
| **14. How do you evaluate your level of knowledge on case-management intervention?** |
| - Very familiar - Somewhat familiar - Not very familiar - Not at all familiar |
| **15. To what extent do you think an intervention is needed or would be useful in your hospital in order to….?** (Likert scale; 0=not useful at all, 10=extremely useful) |
| - Decrease the number of FUEDs emergency room visits - Insure better targeted response to the needs of FUEDs - Support teams facing complex medico-social situations - Spend less time on patients with complicated situations - Spend less time on low-level emergency patients - Facilitate the collaboration with community/primary care |
| **16. To what extent** **do you think a case-management intervention targeting the FUED issue would be useful for your department?**  *Case management is an intervention that consists of coordinating care in collaboration with the patient's healthcare network, while providing individualized support that aims to strengthen their resources and skills (empowerment).* *Case management as a targeted intervention to FUEDs has demonstrated efficiency both in term of reduction of ED attendance and improvement of social background of the patients at the Lausanne University Hospital.* |
| - Extremely useful - Somewhat useful - Not very useful - Not at all useful |
| **17. Do case managers assigned to FUEDs already exist in your hospital?** |
| - Yes - No - I do not know |
| **18. To what extent would you be interested in supporting the implementation of a case management team taking care of FUEDs in your hospital?** |
| - Likert scale (0=not interested at all, 10=extremely interested) |
| **19. To what extent would you be interested in participating to the work of this team?** |
| - Likert scale (0=not interested at all, 10=extremely interested) |

1. *Day, weeks and months were chosen rather than shifts because even shifts are determined on a daily/weekly/monthly basis in Switzerland. Most physicians and nurses do a more or less equal number of days/weeks/months.* [↑](#footnote-ref-1)
