# Supplementary material for: Health care providers’ perception of the frequent emergency department user issue and of targeted case management interventions: a cross-sectional national survey in Switzerland
Source: BMC Emerg Med. 2021 Jan 7;21:4. doi: 10.1186/s12873-020-00397-w (PMC7792123; doi:10.1186/s12873-020-00397-w)
Supplement: Supplementary file 2 — Additional file 2: Appendix 2. Attrition and missing data. [file 12873_2020_397_MOESM2_ESM.docx]

**Appendix 2: Attrition and missing data**

Of the 106 sites targeted, 85 sites responded. Responses that included more than the hospital demographic data was collected from 75 sites (70.7% exploitable responding rate): 48 (63.2%) from the GSP and 27 (90%) from the FISP (21 from the French- and 6 from the Italian-speaking part). Responses were obtained from 12, 14 and 49 ED category 1, 2, 3 respectively. The probability to collect at least one response from a site was significantly higher in the FISP (region x ED category interaction: LRT*χ*^2^=1.53, *P*=0.216; ED category: LRT*χ*^2^= 3.23, *P*= 0.072; region: LRT*χ*^2^= 8.58, *P*= 0.003). The number of responses by ED from which at least one exploitable response was collected was higher in the FISP and decreased from ED category from category 1 to category 3 (region x ED category interaction: 2ΔLogLik=1.13, *P*=0.288; ED category: 2ΔLogLik= 13.56, *P*<0.001; region: 2ΔLogLik=11.31, *P*<0.001).

Overall 208 health professionals (113 physicians and 95 nursing staff) answered the questionnaire: 110 from GSP and 98 from FISP; 42, 62 and 104 from EDs of category 1, 2 and 3 respectively. The number of exploitable responses estimated over 25 items approximated 24.0 ± 0.4 items in questionnaires answered by health professionals from the FISP and 22.6 ± 0.4 items in questionnaires answered by health professionals from the GSP. Missing data was higher in questionnaires answered by health professionals from the GSP than from the FISP (all interactions: LRTχ^2^>2.68, *P*>0.100; profession: LRTχ^2^=0.02, *P* = 0.884; ED category: LRTχ^2^=1.13, *P*=0.287; region: LRTχ^2^=4.93, *P*= 0.026).
