# Supplementary material for: Health care providers’ perception of the frequent emergency department user issue and of targeted case management interventions: a cross-sectional national survey in Switzerland
Source: BMC Emerg Med. 2021 Jan 7;21:4. doi: 10.1186/s12873-020-00397-w (PMC7792123; doi:10.1186/s12873-020-00397-w)
Supplement: Supplementary file 3 — Additional file 3: Appendix 3. Response variables. [file 12873_2020_397_MOESM3_ESM.docx]

**Appendix 3: Response variables**

**Question 6: “***At what frequency have you been confronted with FUEDs over the past two years?”* The answer was coded: every week or day (1) or from never to once or twice a month (0);

**Question 9:** *“How important is the FUEDs problem in your hospital?* The answer “not important” was coded as zero and all other answers (extremely important, important, somewhat important) as one.

**Question 10:** *“How do you evaluate your level of knowledge on FUEDs issue?”* The answers very, somewhat, not very or not at all familiar were coded as 2, 1, -1, -2.

**Question 13:** “*In your opinion, what are the characteristics of FUEDs?”* *Inappropriate use of EDs; High social and medical vulnerability; Suffering from chronic disease; High mortality rates; Patient has no general practitioner; Feeling often discriminated; Often of disadvantaged backgrounds; Presenting psychiatric disorders; Living near an emergency service (<10km); Mostly foreigners.* The characteristics were rated on a 0 to 10 agreement-scale (zero: totally disagree; six: agree; ten: totally agree). The scale of each characteristic was transformed in a -5 to 5 agreement-scale and the scale of the 10 characteristics were averaged in order that zero reflects an absence of agreement, -5 a full misunderstanding and 5 a full understanding of FUEDs characteristics.

**Question 12:** *“Which of these interventions do you know?” Case management, diversion health care strategies (not urgent patients being redirected to other primary health care services), individual health care plan, therapeutic education*. It was assessed using the number of specific interventions targeting FUEDs the professionals know from 0 to 5*.*

**Question 14:**  *“How do you evaluate your level of knowledge on case management intervention?”* The answers very, somewhat, not very or not at all familiar were coded as 2, 1, -1, -2.

**Question 15:** *“To what extent do you think an intervention is needed or useful in your hospital in order to?” Decrease the number of FUEDs' emergency visits; Insure better targeted response to the needs of FUEDs; Support teams facing complex medico-social situations; Spend less time on patients with complicated situations; Spend less time on low-level emergency patients, facilitate the collaboration with community/primary care.* It was rated on a 0 to 10 agreement-scale (zero: totally disagree; six: agree; ten: totally agree). The scale of each utility was transformed in a -5 to 5 agreement-scale and the scale of the 6 utilities was averaged so that zero reflects an absence of agreement, -5: intervention perceived as not useful at all, 5: intervention perceived as extremely useful.

**Question 16: *“****To what extent do you think a case management intervention would be useful in your hospital?”* The answers extremely, somewhat, not very or not at all useful were coded 2, 1, -1, -2.

**Questions 18 and 19:** “*To what extent would you be interested in supporting the implementation of a case management team taking care of FUEDs in your hospital?”* and *“To what extent would you be interested in participating to the work of this team?*” It was rated on a 0 to 10 agreement-scale (zero: totally disagree; six: agree; ten: totally agree). The scales were averaged in order that zero reflects an absence of agreement, -5 a full disinterest and 5 a full interest in CM implementation.
